# Ethnic Differences in Ascending Aorta Dimensions and Dilatation Rates: A Systematic Review

**DOI:** 10.7759/cureus.76703

**Published:** 2024-12-31

**Authors:** Ayub Ansari, Kazi Syed, Mirza Baig, Yazan Tanbour, Abdullah Baghdadi, Aisha Ansari, Ayman Nada

**Affiliations:** 1 Radiology, Kansas City University of Medicine and Biosciences, Santa Clara, USA; 2 Radiology, Kansas City University of Medicine and Biosciences, Kansas City, USA; 3 Radiology, Kansas City University, Kansas City, USA; 4 Radiology, Michigan State University College of Human Medicine, East Lansing, USA; 5 Education, Silicon Valley Academy, Santa Clara, USA; 6 Radiology, Washington University School of Medicine, St. Louis, USA

**Keywords:** aortic diameter, ascending aorta dilatation, cardiology research, cardiovascular health benefits, computed tomography (ct ), ethnic differences, magnetic resonance imaging (mri), personalized medicine (pm), thoracic radiology, transthoracic echocardiography (tte)

## Abstract

Ethnic differences may substantially influence the morphological characteristics of the ascending aorta, with potential implications for clinical assessment and management of aortic dilatation. This systematic review evaluated the impact of ethnicity on ascending aorta diameter and dilatation rates, highlighting the need for more tailored, ethnicity-specific care in cardiovascular practice. We identified 11 studies that measured ascending aorta dimensions using transthoracic echocardiography (TTE), computed tomography (CT), or magnetic resonance imaging (MRI). Most investigations focused on Asian, Caucasian, African American, and Hispanic populations. Data extraction revealed notable variability in baseline aortic diameters across ethnic groups. Some studies found no significant differences between Asian and Caucasian participants, whereas others reported consistently larger diameters in Chinese ethnicities compared to Caucasians and smaller diameters in African American groups. One investigation, for instance, showed that Chinese participants had ascending aorta diameters approximately 1.5 mm larger than their Caucasian counterparts. Dilatation rates also diverged: one study observed that non-White race was linked to earlier or more rapid aortic root dilation in younger populations. At the same time, another reported that Vietnamese individuals had nearly twice the annual growth rate of ascending aorta dilatation when compared to other ethnicities. Available data on prevalence varied, with some studies suggesting ascending aorta dilatation ranged from about 1.2% to 7.5% in Caucasians, 0.9% to 6.4% in African Americans, and 0.8% to 5.9% in Asians. These findings potentiate the role of ethnicity in shaping aortic dimensions, possibly through a combination of genetic predisposition, environmental factors, and lifestyle influences. Incorporating ethnic background into risk stratification may improve the accuracy of clinical assessments and help guide personalized management strategies for ascending aorta dilatation. Future research should address heterogeneity in measurement techniques, more consistently defining ethnic groups, and explore long-term outcomes to clarify whether these observed morphological differences translate into variations in morbidity and mortality.

## Introduction and background

Ascending aortic dilatation is a complex phenomenon that carries a significant risk of life-threatening events such as aortic dissection and rupture when left undetected or unmanaged [1,2]. The thoracic aorta naturally expands over time, with standard reported rates of 0.1 cm per decade in the general population [3]. However, individual variations in dilatation rates are not solely driven by age; numerous studies have demonstrated that genetic predisposition, comorbidities (e.g., hypertension, bicuspid aortic valve), and environmental factors (e.g., smoking) can markedly influence aortic dilation [4,5]. Within this broader scope, ethnicity has emerged as a potential determinant of aortic size and expansion rates, suggesting the need to differentiate management strategies based on population-specific risk profiles [2,6].

Recent work by Mohsin et al. has highlighted a notable discrepancy in ascending aorta diameters between Vietnamese patients and patients from other ethnic backgrounds. In their observational study of 150 individuals undergoing repeated transthoracic echocardiographic (TTE) measurements, Vietnamese participants consistently demonstrated larger mean ascending aorta diameters beyond 60 years of age and double the rate of expansion compared to non-Vietnamese peers despite having a lower mean body mass index (BMI) [7]. Such findings challenge the assumption that smaller body habitus translates to smaller aortic dimensions [8,9]. They also emphasize the importance of understanding the interplay between ethnicity-specific factors-possibly genetic or lifestyle-related-and established risk factors such as hypertension, diabetes mellitus, or obesity [10].

Despite these emerging insights, there remains a paucity of robust data comparing ascending aortic morphology and dilatation across ethnic cohorts. Previous research, including population-based cohorts like the Framingham Heart Study, has offered reference values for aortic dimensions; however, the representation of specific ethnic groups can be limited, and the evidence of cultural or genetic factors remains insubstantial [11]. Given the serious clinical implications of aortic dilatation and the potential for ethnic variability in disease progression, it is imperative to synthesize existing evidence to guide more tailored surveillance and intervention strategies [12]. Therefore, we conducted a systematic review to collate and critically appraise the literature on ethnic differences in ascending aorta diameter and its rate of dilatation. Through this review, we aim to determine whether distinct ethnic groups experience different baseline aortic sizes and expansion rates and elucidate gaps in current data to direct future investigative efforts and inform clinical decision-making.

## Review

Methodology

This systematic review protocol was developed per the Preferred Reporting Items for Systematic Reviews and Meta-Analysis (PRISMA) guidelines. A thorough literature search was conducted using PubMed and Embase from 1 May 2023 to 15 July 2023. The search was performed by five investigators (AA, AA, MB, KS, and YT), who tailored their keywords to each database. The terms "ascending aorta", "dilatation", "diameter", "aortic dilatation", "rate of dilatation", "aortic aneurysm", "ascending aortic aneurysm", "ethnicity", "race", "demographic", "transthoracic echocardiography", "TTE", "computed tomography", "CT", and "magnetic resonance imaging", and "MRI" were combined using the Boolean operators "AND" and "OR" to refine the search results. These keywords were selected to capture studies examining ascending aorta dimensions and dilatation rates with respect to different ethnic backgrounds. Additional articles were identified by reviewing the reference lists of all included studies.

Eligibility Criteria

Studies published in peer-reviewed journals investigating ascending aorta diameter measurements or dilatation rates, with a focus on ethnicity, were included. Both pediatric and adult populations were considered eligible, provided they examined ascending aorta parameters (such as diameter or aneurysm) via imaging techniques, including computed tomography (CT), transthoracic echocardiography (TTE), or magnetic resonance imaging (MRI). Eligible articles were required to compare or report data from multiple ethnic groups (e.g., Caucasian, African, Asian, Hispanic) and to use observational study designs (cross-sectional, cohort, or case-control) or relevant clinical trials reporting primary data. Only articles in the English language were included to ensure consistency in analysis and interpretation.

Studies were excluded if they did not provide specific data on the ascending aorta (e.g. if they focused solely on the descending aorta or aortic arch), did not report ethnicity, or presented insufficient details to determine whether ethnicity was considered as a variable. Editorials, commentaries, or review articles lacking original data were not included. Furthermore, studies with sample sizes of fewer than ten participants, animal studies, in vitro investigations, and cadaveric studies were excluded to maintain a focus on human populations. Non-English language articles were also excluded to enable a coherent synthesis of findings.

Study Selection

Following the database searches, duplicates were removed before screening. Titles and abstracts of the remaining records were initially screened for relevance by six independent investigators. Full texts of potential articles were then retrieved and assessed for eligibility, with any disagreements resolved through discussion or referral to a seventh reviewer.

Data Extraction

Data extraction was performed once the eligible studies were confirmed. The extracted variables included participant demographics (age, gender, and self-reported ethnicity), imaging modalities (CT, TTE, or MRI) used to evaluate the ascending aorta, the actual measurements of ascending aorta diameter, and the documented dilatation rate. Relevant patient characteristics and comorbidities (e.g., aortic dissection, bicuspid aortic valve, Marfan syndrome, hypertension) were also collected if reported. A standardized spreadsheet was used to maintain consistency in data extraction, and any ambiguities were resolved through consensus discussions among the reviewers.

Data Synthesis and Analysis

A narrative synthesis was conducted to evaluate the variations in ascending aorta diameter and dilatation rates among different ethnic groups, given the heterogeneity of study designs, population characteristics, and outcome measures. Findings from transthoracic echocardiography (TTE), computed tomography (CT), or magnetic resonance imaging (MRI) were compared across studies to identify potential associations between age, ethnicity, and ascending aorta dimensions. This review primarily summarizes observed differences in aortic diameter and growth rates among selected ethnic groups. Due to the variability in study methodologies, classification of ethnicity, and clinical heterogeneity across the included studies, a meta-analysis was not feasible. Consequently, a narrative synthesis provides an integrated perspective on how ethnicity may influence ascending aorta parameters, drawing on the collective evidence from the final set of studies.

Results

**Figure 1:**
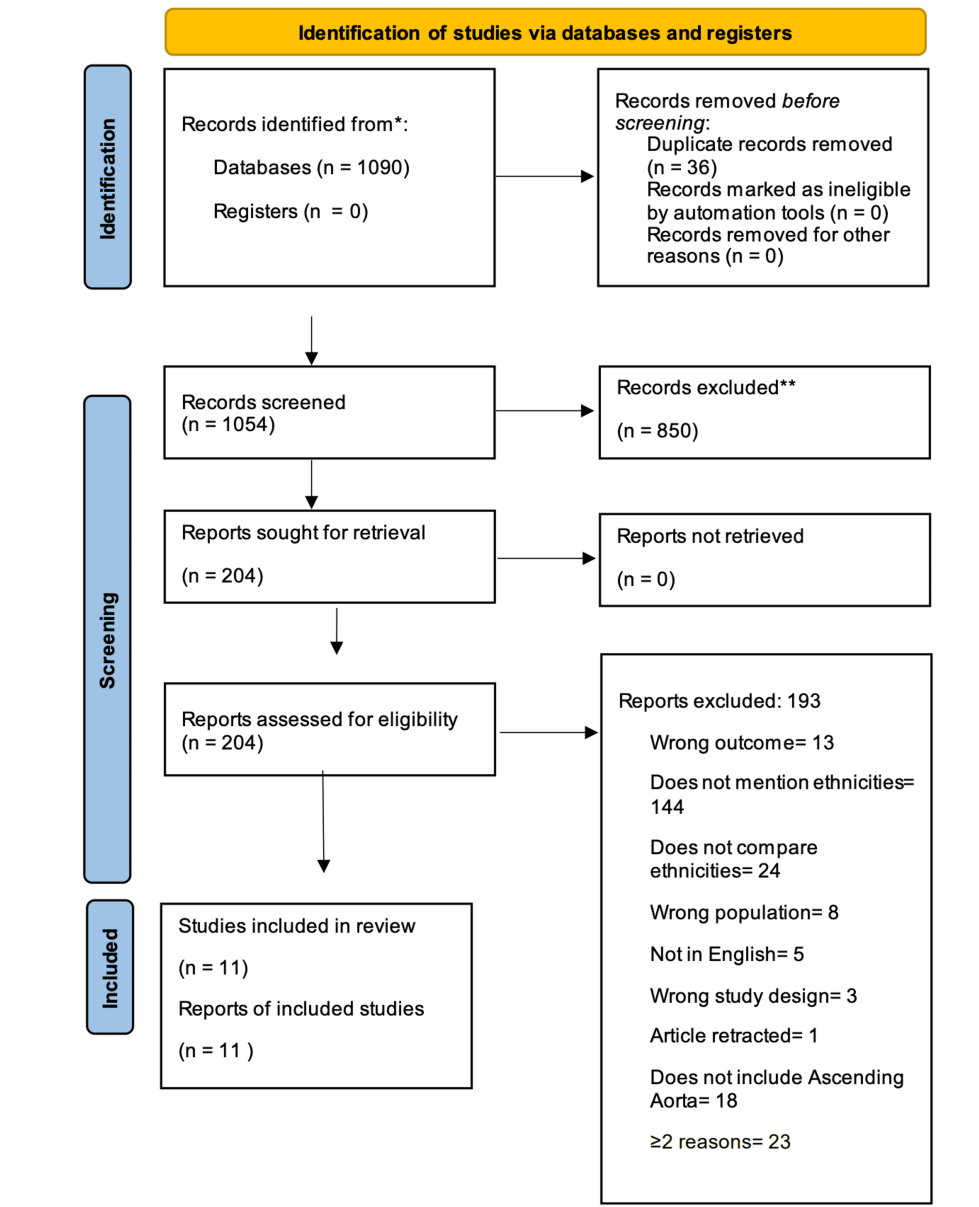
The PRISMA workflow diagram *The number of records identified from the database and register searches. **Any records excluded were excluded by a human; no automation tools were used. PRISMA - Preferred Reporting Items for Systematic Reviews and Meta-Analyses

A total of 1090 records were retrieved from PubMed and Embase, yielding 1054 unique articles after removing 36 duplicates. Of these, 850 were excluded due to a lack of focus on ascending aortic diameter, growth, or related complications or insufficient data on ethnic group comparisons. The remaining 204 articles underwent full-text evaluation, and 193 were subsequently excluded due to issues such as incorrect outcomes, inadequate ethnicity data, or a lack of focus on the ascending aorta. Ultimately, 11 studies met the inclusion criteria and were retained in this review (Figure 1).

A qualitative risk of bias assessment was performed for each included study Table 1. Two reviewers (AA and MB) independently evaluated potential bias in four domains: selection bias (representativeness of participants and inclusion/exclusion criteria), measurement bias (clarity and consistency of aorta measurements), confounding factors (e.g., accounting for age, body surface area, and hypertension), and reporting bias (completeness of outcome reporting). Each domain was judged as "low", "moderate", or "high" risk of bias, and discrepancies were resolved through consensus. Most studies demonstrated a moderate risk of bias due to varying sample sizes, heterogeneous designs, and incomplete confounder adjustment. To enhance the transparency and rigor of our findings, we addressed the PRISMA recommendations, providing a robust assessment of the evidence quality that underpins our conclusions.

**Table 1:** Risk of bias assessment for included studies BSA - body surface area; TTE - transthoracic echocardiography; BP - blood pressure

Study	Selection bias	Measurement bias	Confounding	Reporting bias	Overall risk of bias	Comments
Yoon et al. [13]	Moderate	Low	Moderate	Moderate	Moderate	Sample drawn from a specific hospital-based population. Used CT (low measurement bias). Limited details on controlling confounders.
Chevalier et al. [14]	Moderate	Low	Moderate	Moderate	Moderate	Large sample of young, athletically active individuals (all male). ECHO measurement is standard, but methods to adjust for confounders partially described.
Chiu et al. [15]	Moderate	Moderate	Moderate	Low	Moderate	CT measurements are strong, but small sample (n=120) and limited detail on how subgroups were compared. Confounders (e.g., BSA) not clearly addressed.
Chew et al. [16]	Low	Low	Moderate	Moderate	Low–Moderate	Larger sample (n=562). ECHO indexing to BSA is methodologically robust. Confounding addressed (age, sex, CV risk), but details on data handling are not fully given.
LaBounty et al. [17]	Low	Low	Moderate	Low	Low–Moderate	Very large sample, apparently robust TTE methods. Adjusted for age. Some potential confounders were excluded outright, though not every detail is reported.
Hoskoppal et al. [18]	Moderate	Moderate	High	Moderate	Moderate-high	Pediatric/adolescent population with varied age range. AoR dilation defined by percentile changes; confounding (e.g., growth rates, BSA) might be incompletely addressed.
Turkbey et al. [19]	Low	Low	Low-moderate	Low	Low-moderate	Large multi-ethnic study using MRI (gold standard). Adjusted for hypertension, BP, BSA. Some variables (e.g., genetic predisposition) not fully accounted for.
Vandroux et al. [20]	Moderate	Moderate	Moderate	Moderate	Moderate	Relatively smaller sample (n=513) from one region. TTE standards described. Excluded some conditions. Not all confounders well explained.
Carrero et al. [21]	Moderate	Moderate	Moderate	High	Moderate-high	Article reportedly incomplete. Limited clarity on how population was selected or how outcomes were measured. Possibly incomplete reporting of relevant data.
Rahmani et al. [22]	Moderate	Low-moderate	Moderate	High	Moderate-high	Large sample (n=3152), but article incomplete. Potential for selective reporting; details on adjustments for confounders unclear.
Mohsin et al. [7]	Moderate	Moderate	Moderate	Moderate	Moderate	Moderate sample size (n=150); TTE used to track growth rate. Baseline characteristics reported, but full control for potential confounders not detailed.

Tables 2-4 present the data extracted from the 11 included studies. Table 2 outlines patient characteristics, including sample size, age distribution, gender proportions, and ethnic backgrounds, with some studies focusing on specific ethnic groups such as Caucasians, African Americans, Asians, and Hispanics. Table 3 summarizes ascending aortic measurements obtained through various imaging modalities, including transthoracic echocardiography (TTE), computed tomography (CT), and magnetic resonance imaging (MRI), with some studies indexing measurements to body surface area. Table 4 highlights the presence of relevant pre-existing conditions in each study, such as aortic stenosis, bicuspid aortic valve, Marfan syndrome, and aortic aneurysms, with some studies excluding or separately analyzing these conditions. Despite methodological heterogeneity across the studies, the findings collectively suggest that ethnicity may influence ascending aorta size and expansion rates, while age, gender, and body surface area also demonstrate consistent associations.

**Table 2:** Patient characteristics N/A - not available/applicable; SD - standard deviation

Study	N	Age (Mean ± SD, Min - Max, or Median [IQR])	Gender (%)	Ethnicity/racial background (%)
Yoon et al. [13]	308	81 ± 6.1	Male: 40.9 Female: 59.1	Asian: 65.6, Caucasian: 34.5
Chevalier et al. [14]	336	24 ± 5, range 18–33	Male: 100	Caucasian: 76.4, Afro-Caribbean: 8.8, Pacific Islander: 14.9
Chiu et al. [15]	120	66.8 ± 10.2, range 50–89	Male: 65.0 Female: 35.0	Asian: 100
Chew et al. [16]	562	47 ± 18	Male: 72.0 Female: 28.0	Chinese: 67.0, Malay: 14.0, Indian: 13.0, Eurasian: 5.49
LaBounty et al. [17]	15295	49.9 ± 17.6, range 29–64	Male: 41.3 Female: 58.7	Caucasians: 86.8, Hispanics: 2.46, African Americans: 6.44, Asians: 5.55, Native Americans: 0.25
Hoskoppal et al. [18]	608	11.2 ± 6.3, range 6 months–26	Male: 100	Caucasians: 85.6, African Americans: 7.47, Asians: 2.60, Others: 3.25
Turkbey et al. [19]	3573	60.6 ± 10, range 45–84	Male: 45.7 Female: 54.3	Caucasians: 41.9, African American: 29.6, Chinese American: 11.1, Hispanic: 16.9
Vandroux et al. [20]	513	Male: 40 ± 14, range 30–47	Male: 40.2 Female: 59.9	Sub-Saharan Africans: 100
Carrero et al. [21]	905	38.3 ± 13	Male: 43.8 Female: 56.2	Caucasians: 61.4, Native Americans (Argentinian): 38.6
Rahmani et al. [22]	3152	45–84 (no further detail provided)	Male: 48.1 Female: 51.9	Caucasian: 38.5, African American: 27.8, Chinese American: 11.8, Hispanic: 21.9
Mohsin et al. [7]	150	45–75+ (stratified by age group)	N/A	Vietnamese: 50, Hispanic/Asian (non-Vietnamese)/Caucasian/African American: 50

**Table 3:** Ascending aortic measurements by imaging modality and associations with ethnicity, age, and other factors AA - ascending aorta; AoR - aortic root; AoRd - aortic root dimension; BSA - body surface area; DBP - diastolic blood pressure; I–I - inner‐to‐inner measurement; L–L - leading‐edge‐to‐leading‐edge; MBP - mean blood pressure; NR - not reported; SBP - systolic blood pressure; SS - statistically significant

Study	Measurement methods (CT, MRI, TTE)	Ascending aorta diameter (ethnic group & segment)	Ethnicity findings	Age findings	Preexisting conditions findings	Other relevant factors
Yoon et al. [13]	CT	Mean diameter (mm): • Asian: 30.9 • Caucasian: 30.9 Segment: Ascending aorta	No statistically significant difference	NR	NR	NR
Chevalier et al. [14]	ECHO	• Caucasian: 33 • Afro-Caribbean: 32 • Pacific Islander: 34 Segment: Sinuses of Valsalva	Pacific Islanders had a significantly higher aortic diameter compared to the other groups	Diameter was greater in age >25 years (33.5 mm) than in age 18–25 (32 mm)	NR	High-resistance activities (e.g., forwards) had a larger aortic root (34 mm) vs. backs (32 mm)
Chiu et al. [15]	CT	Mean diameter (mm): • Asian: 39.4 Segment: Ascending aorta	Did not compare with other ethnicities	NR	No recorded chronic health issues that could enlarge the unadjusted diameter	NR
Chew et al. [16]	ECHO	Indexed to BSA (mm/m²): • Chinese: 20.9 • Malay: 20.7 • Indian: 18.4 • Eurasian: 19	Chinese had significantly larger indexed diameters than Eurasians after adjustment; highest dilation rates among Malays	NR	NR	NR
LaBounty et al. [17]	TTE	Males: • White: 32 mm • Black: 30.06 mm • Asian: 31.51 mm • Hispanic: 31.87 mm • Native American: NR Females: • White: 29 mm • Black: 28.21 mm • Asian: 28.78 mm • Hispanic: 28.77 mm • Native American: NR	Black participants had smaller ascending aortas; Asians had larger diameters; differences were statistically significant	Age was corrected for	All comorbidities excluded	NR
Hoskoppal et al. [18]	ECHO	NR	Aortic root (AoR) dilation (change in AoRd/year > 90th percentile) was associated with non-White race	Younger age was associated with a higher AoR dilation rate	NR	AoR dilation was associated with higher sinotubular junction z-score, male sex, and no psychiatric history
Turkbey et al. [19]	MRI	Difference vs. Whites (mm): • Chinese: +1.5 • African American: −0.5 • Hispanic: +0.1 Segment: Ascending aorta	Chinese participants had larger ascending aorta luminal diameters; African Americans had smaller diameters than Caucasians	Age, male sex, and BSA explained ~26% of diameter variability	Hypertension, SBP, DBP, MBP, and PP were positively associated with ascending aorta diameter	NR
Vandroux et al. [20]	TTE	Males I–I end-diastole: 27 mm L–L end-diastole: 30 mm Females I–I end-diastole: 24.5 mm L–L end-diastole: 28 mm Segment: Proximal ascending aorta	Ascending aorta diameters were consistently lower than in Caucasian populations; Africa: 25.7 mm, Europe: 26 mm, US Blacks: 28 mm, Australia: 31 mm, Korea: 29.1 mm	Corrected for age	Excluded in study	No significant sex differences after indexing for BSA
Carrero et al. [21]	TTE	NR	Detected differences in ascending aorta diameter according to ethnicity	Age and BSA were significant determinants of aortic dimension at six levels	NR	Women had smaller absolute TAD due to lower BSA
Rahmani et al. [22]	CT	Indexed AA (mm/m²): • Chinese: 19.8 • Hispanic: 17.8 • Caucasian: 17.6 • African American: 16.6	Highest diameter in Chinese, followed by Hispanics, Caucasians, and African Americans	Diameter increased incrementally with older age groups	NR	Men had larger ascending aorta diameters than women
Mohsin et al. [7]	TTE	Median annual dilatation rate (cm/year): • <60 yrs: 0.025 vs. 0.012 • 61–75 yrs: 0.075 vs. 0.04 • >75 yrs: 0.1 vs. 0.05 (Vietnamese vs. others)	NR	NR	NR	NR

**Table 4:** Pre-existing cardiovascular conditions and comorbidities reported in the included studies AD - aortic dissection; BAV - bicuspid aortic valve; HOCM - hypertrophic obstructive cardiomyopathy; HTN - hypertension; MFS - Marfan syndrome; NR - not reported

Study	Preexisting Conditions (AD, BAV, MFS, HTN, etc.)
Yoon et al. [13]	Aortic stenosis
Chevalier et al. [14]	Among patients with aortic dilation, only one had BAV; some patients had HOCM (not directly relevant to the study)
Chiu et al. [15]	12 normal, 10 with type A dissection, 10 with type B dissection, 14 with aneurysms (4 ascending, 5 descending, 4 multiple locations, 1 arch)
Chew et al. [16]	BAV
LaBounty et al. [17]	None (all comorbidities excluded)
Hoskoppal et al. [18]	Marfan syndrome
Turkbey et al. [19]	42% hypertensive, 10% diabetic, 15% on lipid-lowering medications
Vandroux et al. [20]	None
Carrero et al. [21]	None
Rahmani et al. [22]	None
Mohsin et al. [7]	None

Discussion

The findings of this systematic review reveal noteworthy variations in ascending aorta diameter and dilatation rates among different ethnic groups, reinforcing the notion that ethnicity can play a meaningful role in cardiovascular morphology. Several studies explicitly compared diverse populations, often suggesting that ethnicity can be a meaningful factor in aortic morphology and disease progression. Yoon et al. found no significant difference in ascending aortic diameters between Asian and Caucasian participants, with both groups averaging approximately 30.9 mm [13]. In contrast, Chew et al. observed significantly larger indexed diameters among Chinese participants compared to Eurasians, while Malay participants exhibited higher dilatation rates [16]. Turkbey et al. reported that Chinese participants had ascending aortas approximately 1.5 mm larger than those of White participants, whereas African Americans had aortas about 0.5 mm smaller [19]. LaBounty et al. similarly found that Black participants had smaller diameters compared to White, Asian, and Hispanic individuals [17]. These findings suggest potential ethnic variations in aortic morphology, which may be attributed to a combination of genetic, environmental, and lifestyle factors.

While these cross-sectional measures provide insight into baseline differences, some studies addressed dynamic changes over time. Hoskoppal et al. observed a higher rate of aortic root dilation among non-White participants, particularly in younger individuals [18]. This suggests that certain ethnic backgrounds may be associated with an earlier onset or faster progression of aortic enlargement. Furthermore, Mohsin et al. reported that Vietnamese participants exhibited nearly double the annual growth rate of ascending aortic dilatation compared to individuals from other ethnic groups [7]. These findings highlight the potential influence of ethnicity on the rate of aortic enlargement. It's important to note that this may be due to a complex interplay of genetic, lifestyle, and environmental factors.

Beyond structural and growth-rate disparities, several studies have examined the prevalence of ascending aorta dilatation. While some researchers observed slightly higher rates in Caucasian populations compared to African Americans or Asians, others reported minimal differences after adjusting for confounders such as age, body surface area, and hypertension. For instance, Carrero et al. suggested that demographic variations in aortic measurements could be partially attributed to body surface area, a significant determinant of aortic size [21]. Similarly, Rahmani et al. emphasized the association between older age brackets and larger aortic diameters across all ethnic groups [22]. These findings underscore the intricate interplay between ethnicity and other risk factors, potentially explaining the observed variations in aortic disease susceptibility across different populations.

The majority of studies lacked robust longitudinal data on clinical outcomes, such as aortic dissection, rupture, or mortality. Determining whether observed ethnic differences in aortic diameter or dilatation translate into an increased risk of catastrophic cardiovascular events necessitates future prospective studies with standardized imaging and long-term follow-up. While studies have documented differences across ethnicities, it is crucial to avoid overemphasizing race or ethnicity in isolation. Other significant variables, including comorbidities, social determinants of health, and genetic polymorphisms, must be considered to avoid oversimplification and the potential for perpetuating biases [23].

Despite these caveats, the present findings are relevant for clinical practice. Recognizing that certain ethnic groups may have slightly larger baseline ascending aorta diameters or increased rates of aortic expansion could prompt more nuanced surveillance strategies, particularly for patients near intervention thresholds. For example, some populations might benefit from earlier or more frequent imaging assessments if they exhibit risk factors like hypertension, bicuspid aortic valve, or connective tissue disorders. Still, it remains essential to incorporate ethnicity into a broader context of individualized risk assessment, complementing, rather than replacing, other indicators such as family history, lifestyle, and clinical findings.

Limitations include variability in the definition and reporting of ethnicity, the frequent reliance on hospital-based or regionally limited cohorts, and a predominant focus on Western or developed settings, potentially limiting the generalizability of findings to more diverse global populations. Heterogeneity in imaging modalities and methods for indexing the ascending aorta (e.g., by body surface area) can also hinder direct comparisons across studies. Furthermore, several studies cited in Table 3 and Table 4 either excluded significant comorbidities or did not adequately account for them in analyses, leaving uncertainty regarding the confounding influence of conditions such as hypertension or Marfan syndrome.

These findings suggest that ethnicity may be an important, albeit not exclusive, factor in assessing and managing ascending aortic size and dilatation risk. Further multicenter, longitudinal research - ideally employing standardized measurement techniques and precise definitions of ethnicity - is warranted to elucidate the etiological and clinical significance of the observed ethnic variations [24]. Such investigations would also provide valuable insights into how genetic factors, lifestyle disparities, and healthcare access converge to influence aortic health across diverse populations [17,25,26]. A more nuanced understanding of these interrelationships could ultimately facilitate the development of more personalized and equitable approaches to diagnosing, monitoring, and treating individuals at risk for aortic disease.

## Conclusions

This systematic review highlights both ascending aortic diameter and the rate of dilatation can exhibit significant variability across different ethnic populations. Some studies suggest that Asian populations, including Vietnamese individuals, may demonstrate elevated measurements or faster expansion rates. In contrast, African American cohorts have been observed to have smaller mean diameters in certain studies. While ethnicity appears to be a relevant factor, it is crucial to acknowledge the influence of multiple confounding variables, including age, body surface area, comorbidities, and lifestyle, which substantially impact aortic dimensions and growth. Clinicians should exercise caution when interpreting ethnic differences, ensuring that other key risk factors are carefully considered in personalized assessments. Future prospective, multi-ethnic studies with standardized imaging and rigorous adjustment for confounders are necessary to elucidate the precise role of ethnicity in ascending aortic disease and to optimize monitoring and management strategies for diverse populations.
